# Variable Cre Recombination Efficiency in Placentas of Cyp19-Cre ROSA^mT/mG^ Transgenic Mice

**DOI:** 10.3390/cells12162096

**Published:** 2023-08-18

**Authors:** Prashanth Anamthathmakula, Philemon D. Shallie, Neha Nayak, Sabita Dhal, Jay L. Vivian, Gil Mor, Michael J. Soares, Nihar R. Nayak

**Affiliations:** 1 Department of Obstetrics and Gynecology, University of Missouri-Kansas City School of Medicine, Kansas City, MO 64108, USA; 2 Department of Surgery, University of Missouri Kansas City School of Medicine, Kansas City, MO 64108, USA; 3 Children’s Mercy Research Institute, Children’s Mercy, Kansas City, MO 64108, USA; 4 C.S. Mott Center for Human Growth and Development, Department of Obstetrics and Gynecology, Wayne State University, Detroit, MI 48201, USA; 5 Institute for Reproductive and Developmental Sciences, Department of Pathology and Laboratory Medicine, University of Kansas Medical Center, Kansas City, KS 66160, USA; 6 Department of Obstetrics and Gynecology, University of Kansas Medical Center, Kansas City, KS 66160, USA; 7 Center for Perinatal Research, Children’s Mercy Research Institute, Children’s Mercy, Kansas City, MO 64108, USA

**Keywords:** Cyp19-Cre, placenta, Cre inactivation

## Abstract

The aromatase-Cre recombinase (Cyp19-Cre) transgenic mouse model has been extensively used for placenta-specific gene inactivation. In a pilot study, we observed unexpected phenotypes using this mouse strain, which prompted an extensive characterization of Cyp19-Cre placental phenotypes using ROSA^mT/mG^ transgenic reporter mice. The two strains were mated to generate bi-transgenic Cyp19-Cre;ROSA^mT/mG^ mice following a standard transgenic breeding scheme, and placental and fetal tissues were analyzed on embryonic day 17.5. Both maternal and paternal Cre inheritance were analyzed by mating the respective Cyp19-Cre and ROSA^mT/mG^ males and females. The genotype results showed the expected percentage of Cyp19-Cre;ROSA^mT/mG^ fetuses (73%) and Cre mRNA was expressed in all of the Cyp19-Cre placentas. However, surprisingly, only about 50% of the Cyp19-Cre;ROSA^mT/mG^ placentas showed Cre-mediated recombinase activity as demonstrated by placental enhanced green fluorescent protein (EGFP) expression. Further genetic excision analysis of the placentas revealed consistent results showing the absence of excision of the tdTomato in all of the Cyp19-Cre;ROSA^mT/mG^ placentas lacking EGFP expression. Moreover, among the EGFP-expressing placentas, there was wide variability in recombination efficiency, even in placentas from the same litter, leading to a mosaic pattern of EGFP expression in different zones and cell types of the placentas. In addition, we observed a significantly higher percentage of Cre recombination activity in placentas with maternal Cre inheritance. Our results show frequent mosaicism, inconsistent recombination activity, and parent-of-origin effects in placentas from Cyp19-Cre;ROSA^mT/mG^ mice, suggesting that tail-biopsy genotype results may not necessarily indicate the excision of floxed genes in Cyp19-Cre positive placentas. Thus, placenta-specific mutagenesis studies using the Cyp19-Cre model require extensive characterization and careful interpretation of the placental phenotypes for each floxed allele.

## 1. Introduction

The Cre/loxP system is an integral experimental tool that permits spatial and temporal genetic manipulation during mouse development. The success of conditional gene targeting largely relies on the choice of Cre driver strains, but in the placenta, its use has been limited due to the lack of well-characterized placenta-specific promoters. To date, only a few Cre recombinase-expressing mouse lines have been developed with the ability to target trophoblast lineages in the placenta. Trophoblast-specific Cyp19-Cre was generated by making use of a 501 bp regulatory region within the first exon (I.1) encoding the 5′-untranslated region of human aromatase P450 (CYP19) to drive the expression of the Cre recombinase in the mouse placenta [1]. Upon its initial characterization using ROSA26-LacZ reporter mice, Cyp19-Cre was shown to be active in trophoblast progenitor cells at embryonic day (E) 6.5, and subsequently displayed more widespread activity in spongiotrophoblast, labyrinth trophoblast, and trophoblast giant cells by E10.5-13.5 [1,2,3]. 

The Cyp19-Cre mouse strain has been extensively used for trophoblast-specific disruption of genes to determine gene functions in the placenta. This apparent success has resulted in the determination of the Cyp19-Cre mouse strain as the model of choice for placenta-specific mutagenesis [4,5,6,7,8,9,10,11,12,13]. However, in our preliminary studies, we observed highly unexpected phenotypes using the Cyp19-Cre mouse strain. In a further detailed review of the previously published studies, we also found reports of inconsistent Cyp19-Cre recombinase activity in placentas, including placental cell-type-specific and zone-specific variability and mosaicism [14,15,16]. Although it can significantly impact the interpretation of the results, these variabilities in Cyp19-Cre recombinase activity were not apparent in many studies and were not extensively characterized nor reported. 

In this study, we therefore sought to further characterize the Cyp19-Cre strain. We determined the efficiency of Cyp19-Cre activity in the placenta using ROSA^mT/mG^ reporter mice, which harbor a membrane-targeted tandem dimer Tomato (tdTomato) gene flanked by loxP sites followed by a membrane-targeted enhanced green fluorescent protein (EGFP) cassette [17]. The ROSA26 locus is ubiquitously expressed, enabling the uniform expression of tdTomato in all tissues. Upon Cre-mediated recombination, the tdTomato gene is excised and EGFP expression is activated in cells expressing Cre recombinase [17]. The ROSA^mT/mG^ mouse is a well-characterized double-fluorescent Cre reporter strain known for its robust activity, reliable ubiquitous expression, and breeding performance. Our findings demonstrated dramatic inconsistencies in Cre-mediated loxP-dependent DNA recombination even in the placentas of littermates from pregnant Cyp19-Cre;ROSA^mT/mG^ transgenic mice. 

## 2. Materials and Methods

### 2.1. Animals 

All animal experiments were approved by the Institutional Animal Care and Use Committee (IACUC) at Wayne State University (WSU) and University of Missouri–Kansas City. Mice were housed in a facility controlled for temperature and humidity with a 12 h light–dark cycle. Cyp19-Cre (Tg (Cyp19a1-cre)5912Gle; herein termed as Cyp19-Cre) mice were obtained from Dr. Gustavo Leone [1] and were initially maintained on a FVB genetic background. They were then backcrossed at least nine times onto a C57BL/6J background and maintained on this background for all experiments. ROSA26Sortm4(ACTB-tdTomato,-EGFP) mice, herein known as ROSA^mT/mG^, were obtained from The Jackson Laboratory (stock #007676, Bar Harbor, ME, USA). To determine the efficiency of Cre activity in placentas, Cyp19-Cre animals were bred with the ROSA^mT/mG^ reporter strain, which constitutively expresses tdTomato (mT) in all cells that are replaced by EGFP after Cre-mediated recombination. For maternal inheritance, Cyp19-Cre females (n = 4) were mated with ROSA^mT/mG^ males, while paternal Cre inheritance involved crossing Cyp19-Cre males with ROSA^mT/mG^ females (n = 6). Pregnancies were identified by the presence of a vaginal plug and considered as embryonic day (E) 0.5.

### 2.2. Tissue Collection

Timed pregnant dams were sacrificed at E17.5. The number of implantation sites and resorptions was recorded. Placentas were hemisected in the transverse plane and one half of each placenta was fixed in 4% paraformaldehyde (PFA) for 5 h at 4 °C, cryopreserved in 10–30% sucrose gradient (for 72 h at 4 °C), embedded in OCT (Tissue-Tek, Torrance, CA, USA), and snap frozen on dry ice. The other half of each harvested placenta was minced, flash frozen, and stored at −80 °C for subsequent RNA, DNA, and protein analysis. Genomic DNA was extracted from fetal tail biopsies and genotyped by PCR using the following primers: CYP19-Cre forward primer 5′-GACCTTGCTGAGATTAGATC-3′, reverse primer 5′-GAGAGAGAAGCATGTTTAGCTGGCC-3′; mT/mG wild-type forward primer 5′-AGGGAGCTGCAGTGGAGTAG-3′, mutant forward primer 5′-TAGAGCTTGCGGAACCCTTC-3′, reverse primer 5′-CTTTAAGCCTGCCCAGAAGA-3′; sex-determining gene on the Y chromosome (Sry) forward primer 5′-CCTATTGCATGGACAGCAGCTTATG-3′, and reverse primer 5′-GACTAGACATGTCTTAACATCTGTCC-3′. 

### 2.3. Evaluation of Cyp19-Cre Activity 

Cyp19-Cre mediated recombination in placentas was detected in PFA-fixed 10 µm thick frozen sections via the presence of EGFP-associated fluorescence. Images were captured using a Zeiss fluorescence microscope. Positivity was assessed based on the presence of a green signal in the tissue. Each section was examined/scored by two individuals and grouped either as <30%, 30–70% or >70% Cre activity based on the EGFP signal observed across the placenta.

### 2.4. Gene Excision Analysis and Determination of Recombination

Genomic DNA was extracted from frozen placentas using the DNeasy Blood & Tissue Kit (Qiagen, Germantown, MD, USA) according to the manufacturer’s instructions. For PCR amplification, 60 ng of total template DNA was amplified using forward primer 5′-GTGCTGTCTCATCATTTTGGCA-3′ and reverse primer 5′-TTGCTCACGGATCCTACCTTC-3′. The presence of a 275 bp amplicon indicated Cre-mediated tdTomato gene excision and intra-chromosomal recombination between loxP sites, allowing for EGFP expression. Recombination efficiency was calculated as the percentage of positive placentas (containing the 275 bp amplicon) among the total placentas positive for Cyp19-Cre genotyping.

### 2.5. Reverse Transcription-Quantitative PCR (RT-qPCR)

Total RNA was extracted from frozen placentas using TRIzol (Thermo Fisher Scientific, Waltham, MA, USA). RNA (2 μg) was reverse transcribed into cDNA using an oligo dT primer, and quantitative PCR was performed with the StepOne Plus Real-Time PCR system using a fast SYBR Green PCR Master Mix (Thermo Fisher Scientific, Waltham, MA, USA). Each RT-qPCR reaction was performed in duplicate and the relative expression of Cre mRNA levels (fold change) normalized to Gapdh was calculated using the 2^−ΔΔCT^ method. The primer sequences were as follows: Cre forward primer 5′-ACGAGTGATGAGGTTCGCAAG-3′ and reverse primer 5′-GGTTATTCAACTTGCACCATGCC-3′; Gapdh forward primer 5′-CTTTGGCATTGTGGAAGGGC-3′ and reverse primer 5′-GTGGATGCAGGGATGATGTTC-3′.

### 2.6. Statistical Analysis

Data are represented as mean ± standard error of the mean (SEM). Analyses were performed using GraphPad Prism version 9.0 (GraphPad Software, Boston, MA, USA). Statistical comparisons were carried out using the Student *t*-test or one-way ANOVA followed by post hoc analysis. *p* < 0.05 was considered statistically significant.

## 3. Results and Discussion

### 3.1. Variable Cre Recombinase Activity in Cyp19-Cre;ROSA^mT/mG^ Placentas 

The Cyp19-Cre mouse strain has frequently been used for the disruption of genes in the placenta. However, our preliminary findings and previously published reports indicate that there are inconsistencies in the observed phenotypes across different studies using this Cre strain. We systemically analyzed these inconsistencies in placentas using the Cyp19-Cre and ROSA^mT/mG^ transgenic mice. We used the ROSA^mT/mG^ Cre reporter mouse because of its known robust activity, reliable ubiquitous expression, and breeding performance, as demonstrated in previous research [17]. Additionally, in a preliminary study using the LysMcre;ROSA^mT/mG^ transgenic mice, we observed the expected and highly consistent EGFP expression in macrophages at the maternal–fetal interface (Supplemental Figure S1). Several previous studies have also utilized the ROSA^mT/mG^ reporter strain to characterize placental Cre recombinase activity [18,19,20].

**Figure 1:**
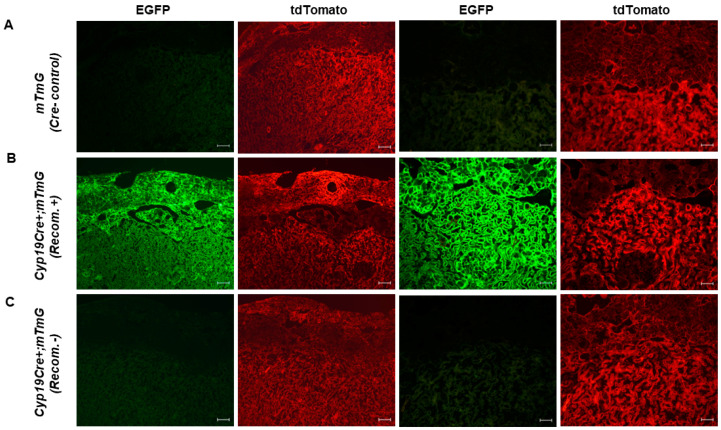
Placenta-specific expression of Cre activity in Cyp19-Cre;ROSA^mT/mG^ transgenic mice. Cyp19-Cre recombinase activity was assessed via EGFP fluorescence of E17.5 placental cryosections. Representative low (panels 1 and 2) and high-magnification (panels 3 and 4) images of (**A**) ROSA^mT/mG^ control, (**B**) recombination-positive Cyp19-Cre;ROSA^mT/mG^ and (**C**) recombination-negative Cyp19-Cre;ROSA^mT/mG^ placentas. In recombination-positive Cyp19-Cre;ROSA^mT/mG^ placentas, a robust positive EGFP signal in the labyrinth and junctional zones was observed, while recombination-negative Cyp19-Cre;ROSA^mT/mG^ placentas were completely devoid of EGFP positive cells. tdTomato shows penetrance of expression in all placental cells. Scale bars in panels 1 and 2 are 100 μm. Scale bars in panels 3 and 4 are 50 μm. mTmG (Cre- control) = ROSA^mT/mG^ placenta, Cyp19Cre+;mTmG (Recom.+) = recombination-positive Cyp19-Cre;ROSA^mT/mG^ placenta, Cyp19Cre+;mTmG (Recom.-) = recombination-negative Cyp19-Cre;ROSA^mT/mG^ placenta.

Initially, to confirm Cre activity in trophoblast cells, female Cyp19-Cre transgenic mice were mated with ROSA^mT/mG^ reporter male mice. We assessed EGFP fluorescence, which is indicative of Cyp19-Cre activity, in E17.5 placentas positive for Cyp19-Cre genotyping and containing the mT/mG allele. Compared to their wild-type littermates (ROSA^mT/mG^), Cyp19-Cre;ROSA^mT/mG^ presented a robust, positive EGFP signal in the labyrinth and junctional zones of some placentas (Figure 1A,B), which is consistent with the original report [1,2]. We also observed a positive EGFP signal in the upper decidual zone adjacent to the junctional zone (Figure 1B), which is in line with several previously reported studies [8,14,21]. A further immunohistochemical analysis for Cytokeratin-8 (CK8) revealed that the EGFP signals likely came from the invading trophoblast cells in the upper decidual region (Supplemental Figure S2) [22,23]. However, surprisingly, some Cyp19-Cre;ROSA^mT/mG^ placentas from the same pregnant dam were completely devoid of EGFP-positive cells, indicating an absence of Cre activity in placentas carrying the Cyp19-Cre allele (Figure 1C).

**Figure 2:**
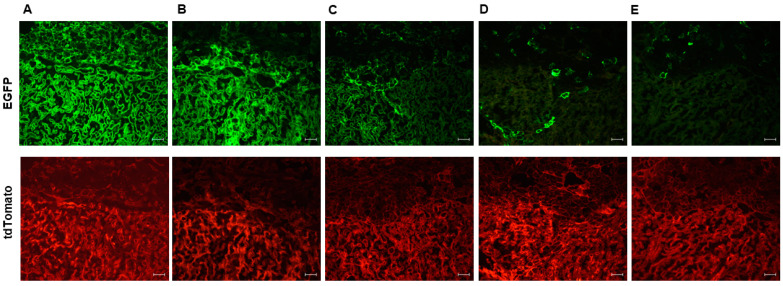
Mosaic activity of Cre in Cyp19-Cre;ROSA^mT/mG^ transgenic mice. Representative images of E17.5 placental sections with EGFP indicating Cyp19-Cre activity in (**A**) labyrinth and junctional zone, (**B**) predominantly and (**C**) partially in the labyrinth. Sporadic EGFP-positive cells were detected in (**D**) both labyrinth and junctional zone or (**E**) junctional zone alone. Note the mosaic and inconsistent EGFP expression in different zones and cell types of the placentas from the same litter. tdTomato shows penetrance of expression in all placental cells. Scale bars in images are 50 μm.

These findings prompted us to examine the Cre activity in all 99 Cyp19-Cre;ROSA^mT/mG^ genotype-positive placentas (out of a total of 137 placentas) from 16 different litters, irrespective of maternal or paternal Cre inheritance. Unexpectedly, we observed an extreme mosaic pattern in some placentas and a complete lack of EGFP expression in other Cyp19-Cre;ROSA^mT/mG^ genotype-positive placentas, irrespective of the mating scheme. Figure 2 shows differential Cre activity ranging from a robust EGFP signal in both labyrinth and junctional zones (Figure 2A) to a pattern restricted primarily to the labyrinth zone (Figure 2B). We also observed the heterogeneity of EGFP fluorescence within the labyrinth zone (Figure 2C), as well as the random appearance of EGFP-positive cells in both labyrinth and junctional zones (Figure 2D) or in the junctional zone alone (Figure 2E). Such observations were not previously reported, and these data provide strong evidence that the detection of the Cyp19-Cre allele in tail biopsies (genotyping) may not always drive the excision of floxed genes in the placenta and that variable Cre recombinase activity can be displayed between littermates. 

Consistent with our findings, it has been previously reported that the labyrinth zone trophoblast cells of E16 placentas exhibit mosaic Cyp19-Cre recombinase activity using the Rosa26 tdTomato reporter mouse strain [15]. In addition, it has also been found that Cyp19-Cre recombinase activity varies in different reporter mouse strains. Moreau et al. used two different reporter mice, Z/EG and ROSA26-LacZ, to analyze Cyp19-Cre activity in E14.5 placentas and found that the Z/EG strain had more diverse and widespread Cre recombinase activity than the ROSA26-LacZ strain [14]. Based on these findings, the authors suggested that the Rosa26 locus may not be expressed in all placental cell types or may not be accessible for the Cre-mediated excision of loxP sites [14]. However, a recent study has revealed that there is varying Cyp19-Cre activity in two Rosa26 mouse reporter lines. The Cyp19-Cre activity was found to be strong in the junctional zone and trophoblast cells in the labyrinth zone of E16 placentas in the Rosa26 tdTomato reporter mouse line, whereas placentas from the Rosa26-EYFP reporter line showed inconsistent and weak Cre activity [16]. In E12.5 placentas, Shawber et al. reported predominant Cyp19-Cre activity in trophoblast cells of the junctional zone and labyrinth using ROSA26 tdTomato and ROSA26-LacZ reporter mice [8]. Akhaphong et al. validated the Cyp19-Cre activity in E17.5 placentas using CAG-ZsGreen1 reporter mice and reported GFP expression throughout the entire placenta [11]. Other studies have also reported on the specificity of the Cyp19-Cre activity using ROSA26-EYFP mice in E17.5 placentas [24]. There are several factors that can impact the expression of Cyp19-Cre, such as the genetic background, chromatin accessibility of loxP sites, promoter strength, or epigenetic changes. However, the reason behind the inconsistent activity of Cyp19-Cre across various reporter mice in previous studies remains unclear. Our findings suggest that the primary reason for the varying Cyp19-Cre activity reported in past studies is likely due to the extensive mosaicism or complete lack of Cyp19-Cre recombinase activity in the placentas of littermates. To ensure valid conclusions, future studies involving Cyp19-Cre mice must thoroughly characterize each floxed allele.

### 3.2. Lack of Cre-Mediated Recombination at loxP Sites in Cyp19-Cre;ROSA^mT/mG^ Placentas 

**Figure 3:**
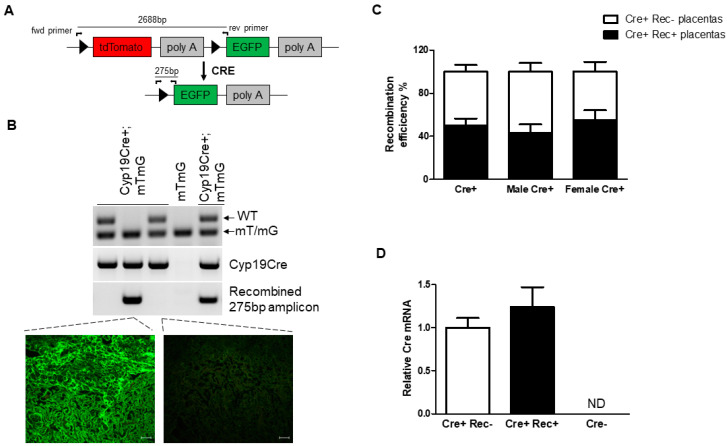
Absence of Cre activity in E17.5 littermates in Cyp19-Cre transgenic mice. (**A**) Schematic representation of excision of loxP sites by Cre recombinase. Triangles represent loxP target sites and the primer binding sites are represented by black arrows. (**B**) Representative PCR genotyping for the presence of the mT/mG and Cyp19-Cre transgenes in littermates. Lower gel image shows the presence of recombined allele in two of the littermates (columns 2 and 5) and absence of recombination (columns 1 and 3). The placentas in wells 2 and 3 were analyzed for EGFP expression. Note the presence and absence of EGFP expression in these placentas. Scale bars in images are 50 μm. (**C**) Recombination efficiency in Cyp19-Cre;ROSA^mT/mG^ placentas. No significant gender-specific effects on recombination efficiency between male and female offspring were observed. (**D**) RT-qPCR showing relative Cre expression in recombination-positive and recombination-negative Cyp19-Cre;ROSA^mT/mG^ transgenic mice. Cre mRNA levels were found to be similar between recombination-positive and recombination-negative placentas, while no Cre expression was observed in ROSA^mT/mG^ (Cre negative) placentas. All graphs represent mean ± SEM. WT = wild type, mT/mG = transgene, Cre+ Rec− = recombination-negative Cyp19-Cre;ROSA^mT/mG^ placenta, Cre+ Rec+ = recombination-positive Cyp19-Cre;ROSA^mT/mG^ placenta, Cre− = ROSA^mT/mG^ placenta.

The absence of EGFP expression in Cyp19-Cre;ROSA^mT/mG^ placentas could suggest that the loxP sites in the tdTomato gene were not being excised, resulting in intra-chromosomal recombination failure. To provide a readout of recombination efficiency, we verified genetic excision by designing primers that bind regions outside the loxP sites (Figure 3A, schematic). Recombination at the loxP sites results in the excision of the tdTomato gene and generates an amplicon of 275 bp, confirming DNA excision. We analyzed the genomic DNA from Cyp19-Cre;ROSA^mT/mG^ placentas (n = 99) isolated from 16 pregnant females. The ROSA^mT/mG^ transgenic mice were identified by PCR genotyping (primer sets recommended by the Jackson Laboratory), where either a double band for heterozygotes (WT and mT/mG transgene) or a single band for homozygotes (mT/mG transgene) was observed (Figure 3B, top panel). Next, we analyzed the presence of the Cyp19-Cre transgene, which gives rise to an amplicon of 545 bp (Figure 3B, middle panel) based on primers described in the original paper for characterizing the Cyp19-Cre transgenic mice. Genetic excision analysis revealed the presence (columns 2 and 5) and absence (columns 1 and 3) of recombination (275 bp amplicon) in a subset of placentas within a litter collected from the same pregnant female (Figure 3B, bottom panel). Accordingly, we observed the presence and absence of EGFP expression in these placentas. We used ROSA^mT/mG^ placentas in the absence of Cre as negative controls for recombination. Upon calculation of the recombination efficiencies, we observed that only 50% (49 out of 99) of Cyp19-Cre;ROSA^mT/mG^ placentas (pregnant females, n = 16) showed excision of the tdTomato gene, while the rest were negative for recombination (Figure 3C). We did not observe any significant gender-specific effects on recombination efficiency between male and female offspring, being 43% and 57%, respectively (Figure 3C). Taken together, these results confirm the inconsistent recombination within littermates and support the findings from the EGFP immunofluorescence analysis (Figure 1 and Figure 2). To elucidate the absence of recombination and Cre activity, we examined the levels of Cre mRNA in all experimental samples. While Cre expression was not observed in the ROSA^mT/mG^ (Cre negative) placentas, Cre mRNA levels were found to be similar between recombination-positive and negative placentas, suggesting that Cre inactivation is not occurring at the transcriptional level (Figure 3D). 

### 3.3. High Level of Recombination Efficiency with the Maternally Inherited Cre Allele in Cyp19-Cre;ROSA^mT/mG^ Placentas 

**Figure 4:**
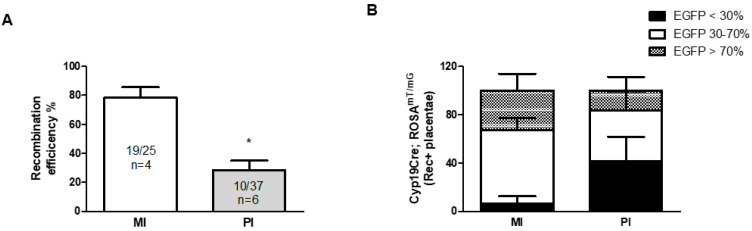
Recombination efficiency of Cyp19-Cre in vivo. (**A**) Genomic analysis showing Cre-mediated tdTomato gene excision in placentas that inherited Cyp19-Cre transgene either maternally (MI) or paternally (PI). A higher percentage of Cre recombination efficiency in placentas with maternal Cre inheritance was observed. (**B**) EGFP expression observed as percentage of placental area in recombination-positive Cyp19-Cre; ROSA^mT/mG^ placentas. Majority of the placentas had variable EGFP expression (i.e., ≤70% EGFP signal). * *p* < 0.05 student *t*-test. All graphs represent mean ± SEM. Rec+ = recombination positive.

We analyzed the recombination efficiency of placentas derived from either the maternal or paternal inheritance of the Cyp19-Cre allele. Figure 4A shows variable Cre activity in Cre placentas depending on the parent of origin. Maternal inheritance resulted in a recombination efficiency of 76% (19 out of 25; n = 4 pregnant females), while paternal inheritance had a recombination efficiency of only 27% (10 out of 37; n = 6 pregnant females). The aromatase-based Cre driver is not an imprinted gene, and although we observed variable EGFP expression in placentas among Cyp19-Cre;ROSA^mT/mG^ littermates (Figure 1 and Figure 2), our observations are in line with the original study of the Cyp19-Cre transgenic mouse conducted by Wenzel and Leone [1]. They reported a more consistent and intense expression of Cyp19-Cre throughout the placenta when it was maternally inherited. Furthermore, several studies have also indicated parent-of-origin effects for other non-imprinted genes [25,26,27]. 

We also analyzed the intensity of the EGFP signal in all recombination-positive Cyp19-Cre;ROSA^mT/mG^ placentas due to the variable Cre recombinase activity exhibited by the Cyp19-Cre allele. Our findings revealed that only 33% of the placentas with maternal Cre inheritance had more than 70% Cre activity as evaluated by EGFP fluorescence (Figure 4B). A majority of placentas (61%) had EGFP fluorescence ranging from 30–70% (Figure 4B). This is consistent with a recent study by Sandovici et al. on the role of IGF2 in placental development, where only ~23–29% of all KO mutants (Cyp19-Cre;Igf2^fl/fl^;Rosa26-EYFP) had more than an 80% deletion of the Igf2 floxed allele [16]. However, in another report, Dicer KO in the trophoblast lineage which was generated using the maternally inherited Cyp19-Cre transgene, did not result in the significant deletion of the floxed Dicer allele [28]. When paternally inherited, our results showed that only about 17% of Cre placentas had widespread robust Cyp19-Cre activity (>70%) as determined by an EGFP signal, and 42% of placentas had less than 30% EGFP fluorescence. Other studies have also reported that paternally inherited Cyp19-Cre can affect excision efficiency, with Wieczorek et al. [21] reporting that only around 30% of the placentas had a widespread signal. 

## 4. Conclusions

In our study using ROSA^mT/mG^ reporter mice, we found that the Cyp19-Cre driver strain exhibits inconsistent Cre recombination activity and varying levels of mosaicism and parent-of-origin effects in the placenta, even among littermates. Although Cre-mediated recombination efficiency is influenced by both the Cre driver and the floxed target allele, our work provides compelling evidence of the extensive variability of Cyp19-Cre-mediated recombination activity in the placenta, which was not clearly delineated in previous reports. Our findings also indicate that relying solely on the genotyping analysis to interpret results from the Cyp19-Cre model can lead to highly misleading conclusions. Therefore, it is crucial for future studies to thoroughly characterize the Cyp19-Cre model for each floxed allele and carefully interpret the placental phenotypes.

## Data Availability

Data is contained within the article and Supplementary Materials.

## Supplementary Materials

The following supporting information can be downloaded at: https://www.mdpi.com/article/10.3390/cells12162096/s1.
