# Supplementary material for: Variable Cre Recombination Efficiency in Placentas of Cyp19-Cre ROSAmT/mG Transgenic Mice
Source: Cells. 2023 Aug 18;12(16):2096. doi: 10.3390/cells12162096 (PMC10453067; doi:10.3390/cells12162096)
Supplement: Supplementary file 1 [file cells-12-02096-s001.zip › cells-2503656-supplementary.pptx]

## Slide 1
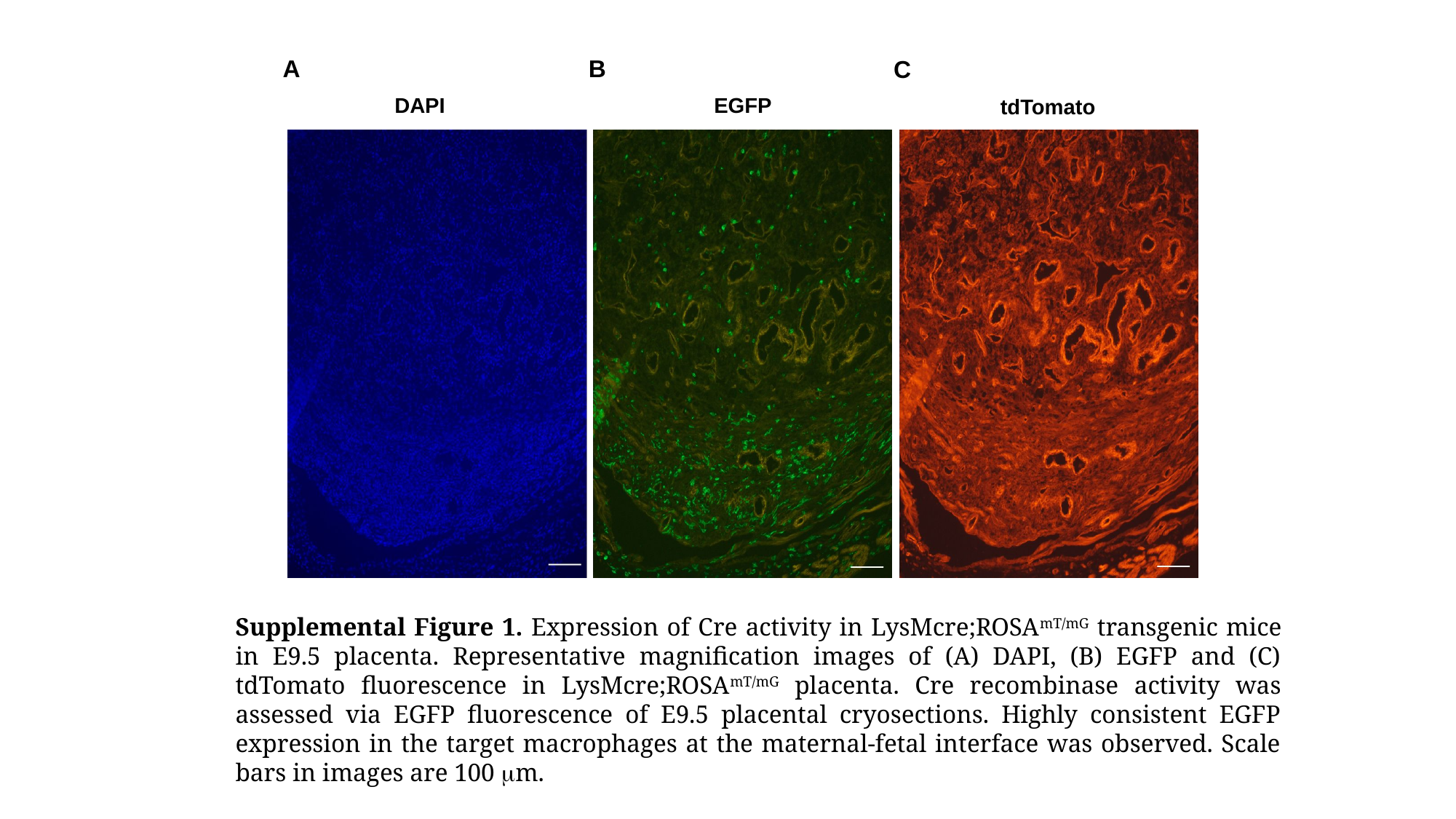

A
 B
C
DAPI
EGFP
tdTomato
Supplemental Figure 1. Expression of Cre activity in LysMcre;ROSAmT/mG transgenic mice in E9.5 placenta. Representative magnification images of (A) DAPI, (B) EGFP and (C) tdTomato fluorescence in LysMcre;ROSAmT/mG placenta. Cre recombinase activity was assessed via EGFP fluorescence of E9.5 placental cryosections. Highly consistent EGFP expression in the target macrophages at the maternal-fetal interface was observed. Scale bars in images are 100 μm.

## Slide 2
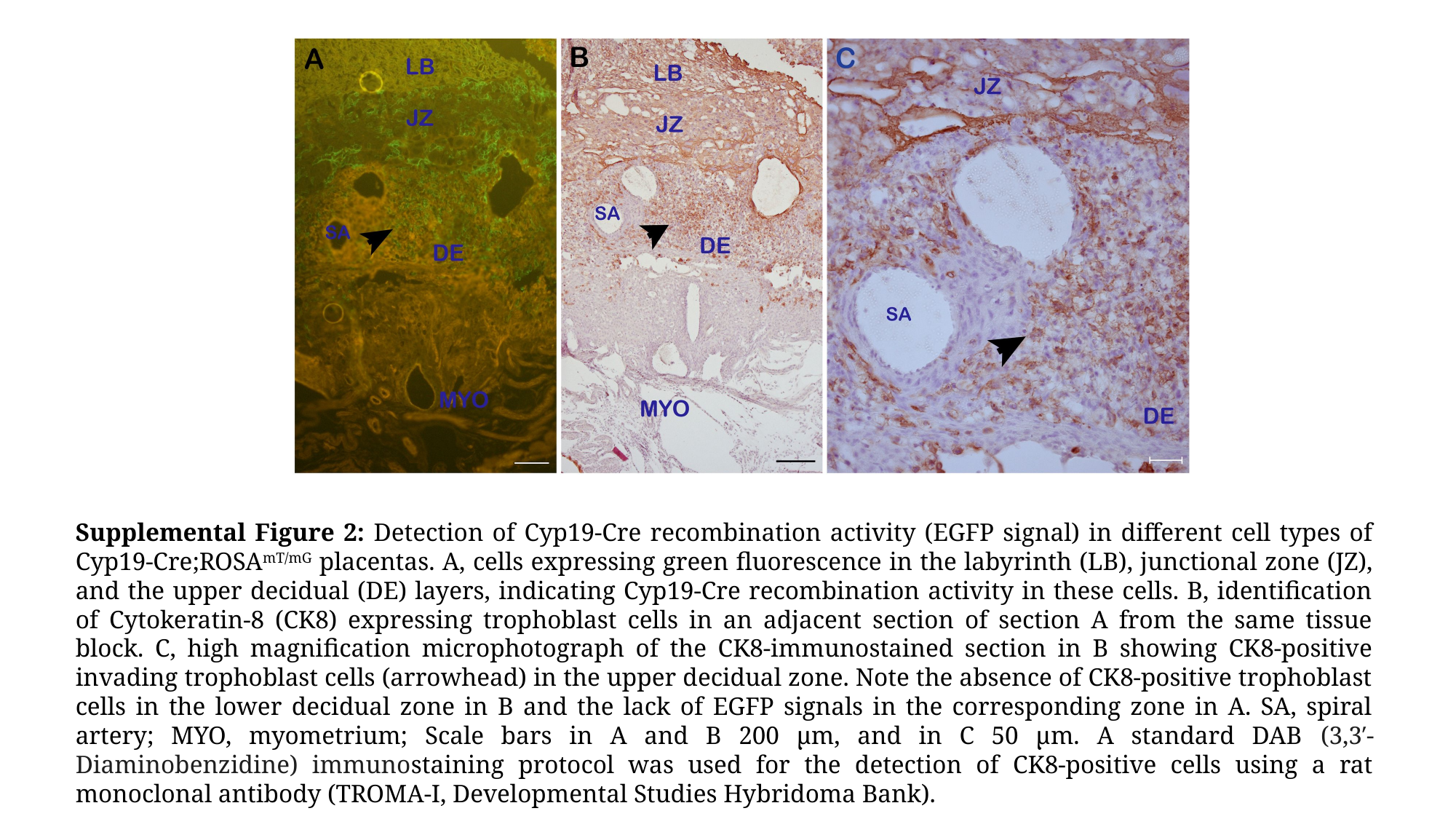

Supplemental Figure 2: Detection of Cyp19-Cre recombination activity (EGFP signal) in different cell types of Cyp19-Cre;ROSAmT/mG placentas. A, cells expressing green fluorescence in the labyrinth (LB), junctional zone (JZ), and the upper decidual (DE) layers, indicating Cyp19-Cre recombination activity in these cells. B, identification of Cytokeratin-8 (CK8) expressing trophoblast cells in an adjacent section of section A from the same tissue block. C, high magnification microphotograph of the CK8-immunostained section in B showing CK8-positive invading trophoblast cells (arrowhead) in the upper decidual zone. Note the absence of CK8-positive trophoblast cells in the lower decidual zone in B and the lack of EGFP signals in the corresponding zone in A. SA, spiral artery; MYO, myometrium; Scale bars in A and B 200 µm, and in C 50 µm. A standard DAB (3,3′-Diaminobenzidine) immunostaining protocol was used for the detection of CK8-positive cells using a rat monoclonal antibody (TROMA-I, Developmental Studies Hybridoma Bank).
